# Decomposition Analysis of Antenatal Care Utilization Inequities in Kembata Tembaro Zone, Southern Ethiopia

**DOI:** 10.5334/aogh.4101

**Published:** 2023-10-20

**Authors:** Dejene Ermias Mekango, Sisay Moges, Bereket Abrham Lajore, Alula Seyum Buda, Tekle Ejajo, Desta Erkalo

**Affiliations:** 1 Public Health Department, College of Medicine & Health Sciences, Wachemo University, Hosanna, ET; 2 Department of Family Health, Hosanna College of Health Science, Hosanna, ET; 3 School of Nursing, College of Medicine & Health Sciences, Wachemo University, Hosanna, ET

**Keywords:** decomposition, inequities, antenatal care, Southern Ethiopia

## Abstract

**Background:**

Health equity has emerged as a global issue in the post-2015 Sustainable Development Goals, and Ethiopia is no exception. Despite positive improvements, inequities in maternal health service utilization among demographic groups continue to be one of Ethiopia’s significant challenges in decreasing maternal mortality. This study focuses on antenatal care service discrimination among a local poor group known as the “golden hands” community in Ethiopia’s Kembata Tembaro zone. The subgroup community consists of outcast artesian groups known as “golden hands,” formerly known as “Fuga,” who face discrimination in all aspects of life owing to their living conditions and ethnic background.

**Methods:**

A community-based comparative cross-sectional study was conducted in Ethiopia’s Kembata Tembaro, zone in the Southern Nations, Nationalities, and Peoples’ Region (SNNPR), from January to February 2022. The study focused on two groups, “golden hands” and “non-golden hands,” consisting of women aged 15–49 years. Using stratified and multistage cluster sampling, 1,210 participants were selected, with 440 from golden hand communities and 770 from non-golden hand communities. Data was collected through translated questionnaires, and data quality was rigorously monitored. The concentration curve and index, as well as logistic-based decomposition analysis, were used to examine inequality. The statistical significance threshold was set at *p* < 0.05 with a 95% confidence interval.

**Result:**

This study comprised 1,210 eligible participants, 440 of whom were golden hand community members. Discrimination accounted for 60.23% of the decreased antenatal care (ANC) service use by the golden hand community. Age, urban residence, and wealth index were the most important independent factors with statistically significant contributions to changes owing to differences in effects (discriminated difference).

**Conclusion:**

Since ANC service discrimination is prevalent, the government and nongovernmental organizations should take steps to ensure that marginalized groups in society, such as golden hand women, the poor, the uneducated, and rural people, have equal access to service utilization opportunities.

## Introduction

Globally, there are growing inequalities concerning health and health care delivery [1,2,3], and to help reduce these inequities, nations around the world are in the pursuit of achieving universal health coverage. However, several nations emphasize their efforts in reducing national mortality and morbidity rates over resolving inherited issues caused by imbalances in health care systems [4]. For instance, every day, more than 800 women are estimated to die from complications related to pregnancy and delivery throughout the world due to low prenatal coverage, with sub-Saharan Africa accounting for the majority of deaths [5,6].

Furthermore, inequities in health include not only the unequal distribution of health but also unjust distribution of health as a result of unfair or insufficient social systems [7]. Health disparities result from a systematically uneven allocation of power, prestige, and resources among societal groups [8]. Evidence suggests that women and children in disadvantaged demographic segments have lower preventive treatment coverage and usage as well as poorer health outcomes than more advantaged populations [9,10]. These discrepancies are serious public health problems with social and economic ramifications [9,10,11]. Access to equitable quality maternal health care is critical to improving maternal health outcomes and is extensively advocated for. In low-income nations, disparities in residential location (urban/rural), household affluence, and the mother’s educational status are identified as important barriers to obtaining high coverage [12,13]. Thus, health equity has become a worldwide goal in the post-2015 Sustainable Development Goals (SDGs), and it is also a focus in Ethiopia [14].

Ethiopia has implemented a pro-poor health policy through a variety of health programs, including the Health Extension Program, the Health Development Army, and the establishment of community-based health insurance schemes aiming to enhance maternal health usage services [15,16]. The health care that a woman receives throughout her pregnancy is critical for both the mother’s and her baby’s survival and well-being [5]. Antenatal care (ANC) provides the chance to provide appropriate therapy to reduce low birth weight and boost newborn survival [17].

In Ethiopia, ANC coverage has significantly increased over the previous two decades [17]. Despite the progress, significant disparities in health outcomes persist due to variations in economic position, education, location of residence, and gender [4]. Maternal health service consumption discrepancies among demographic groups continue to be a challenge in decreasing maternal mortality in Ethiopia. Inequities in maternal health services consumption were growing in 2016 compared to 2000, with low socioeconomic status, illiteracy, rural location, lack of employment, and lack of access to mass media being the main factors [15]. Access to maternal health care services remains a significant problem in low-income areas [18]. Even though maternal health services are provided free of charge in Ethiopia, the full attendance of ANC is about 62%, and attendance is much better in urban areas than in rural areas [19].

This study focuses on the disparities in ANC utilization among a local underprivileged population known as the “golden hands” community. In the Kembata Tembaro zone, there are outcast artisan groups known as “Fuga” who are akin to the “untouchables” in India. Kambati Menti Gezima (KMG) Ethiopia is striving to improve the social and economic situations of these villages with the support of USAID project monies using the tried-and-true tactics of community discourse and social mobilization. KMG has collaborated with both populations to uncover the root causes of prejudice and exclusion [20]. The study addressed existing equity gaps in which to intervene, and evaluating the interdependence of multiple factors of health disparities may be a significant component in understanding and acting on socially and economically disenfranchised women. As a result, the purpose of this study will be to assess differences in ANC service inequity depending on community groupings such as the golden hand and non-golden hand strata.

## Methods and Materials

### Study design and setting

A community-based comparative cross-sectional was conducted in Ethiopia’s Kembata Tembaro zone, SNNPR, from January 3 to February 27, 2022.

### Study population

The study population was divided into two categories: golden hand and non-golden hand. The study population included all women of reproductive age residing in selected kebeles (small administrative units) representing non-golden hand communities across the zone, as well as women from golden hand communities throughout the zone. The study included women of reproductive age (15–49 years) who had lived in the study areas for at least six months and had at least one child. Women were excluded if they were unable to participate due to illness and had a known history of infertility at the time of data collection.

### Sample size and sampling procedures

To estimate sample size, Epi info version 7 was used. The following hypotheses were used: The concentration index (CI) of inequity in the utilization of maternal health care was 13.2, 17.5, and 14.1 percent, respectively; the service is highly favored by the economically better off [21], 95% (confidence interval), power of 80%, and design effect of two because the sampling technique uses multistage sampling; and 10% nonrespondent rate was also included. The sample size was calculated using the same assumptions for all outcome variables and the second objective, and the maximum sample size was taken as the minimum required sample size, which is 880 (440 golden hand vs. 440 non-golden hand). However, all eligible participants in the chosen cluster were interviewed; therefore, a total of 1,210 (440 golden hand vs. 770 other non-golden hand community) study participants were included in the study.

The subjects for the study were chosen using stratification (stratified sampling) based on population characteristics, followed by a multistage cluster sampling method. The study’s participants were split into two groups: the golden hand community and the non-golden hand community. The study then enrolled all members of the golden hand community who met the inclusion criteria, regardless of where they lived, for a total sample size of 440. A multistage cluster sampling approach was used to sample members of the non-golden hand community. In the first stage of cluster sampling, more than 20% of the nine districts (woredas) in the Kembata Tembaro zone were chosen, resulting in the selection of four districts (woredas). In the second stage of cluster sampling, more than 20% of kebeles from each selected woreda were included in the study, totaling eight kebeles with similar sociodemographic characteristics as the golden hand community subgroup final administrative unit in the sampling process (two from each woreda). As a result, from the households in the kebeles described in the study, 770 women between the ages of 15 and 49 who had at least one child in a non-golden hand community were chosen.

## Data collection instruments, procedures, and data quality control

The survey instruments were adapted from the Demographic and Health Survey and the WHO framework for measuring health equity [8,22]. Data was collected using the open data kit (ODK) electronic data collection platform. We used a structured interviewer-administered questionnaire. Sixteen data collectors with a minimum of grade 12 and the ability to communicate in the local language, as well as eight nurses with a supervisory diploma, were recruited and assigned as data collectors and supervisors, respectively.

To ensure the consistency of the questionnaire, the English version of the instrument was translated into the local language (Kembatissa) and then retranslated into English by independent translators. Before the actual data collection time, a pretest was conducted in 10% of the sample size outside of the study population with similar socioeconomic backgrounds. Significant changes and a logical flow of ideas were implemented based on the pretest results. The principal investigators trained them for two days on the study’s objectives, including how to select households; conduct interviews, including consent-taking methods; and handle data. Supervisors reviewed and checked the questionnaires for completeness and relevance every day, and all necessary feedback was provided to data collectors the following morning before data collection.

Participants’ overall knowledge on ANC was classified as “good” if the score was between 70% and 100% (7–10 points), “moderate” if the score was between 50% and 69% (5–6 points), and “poor” if the score was less than 50% (0–4 points). Attitude toward ANC was assessed using seven questions and graded on a 5-point Likert scale. The overall level of attitude was categorized using modified Bloom’s cutoff points as positive if the score is 80%–100% (≥ 20 points), neutral if the score is 60%–79% (15–19 points), and negative if the score is less than 60% (<15 points).

### Measurement of inequities

In this study, inequity is measured using concentration curve and concentration index. Concentration indices are frequently used to measure inequality in one variable over the distribution of another. To estimate equity for ANC utilization, four standard equity measures—equity gaps, equity ratios, concentration curves, and concentration indices based on community grouping—were used [3,23]. First, we examined differences in coverage for the outcome variable and explanatory variables by community subgrouping using bivariate statistics. To determine equity, we used simple comparative rates/measures of coverage for two groups [24]. Furthermore, when the wealth quantile was taken into account, the concentration index and concentration curve were used to demonstrate the inequity and difference in service utilization between the two groups.

### Concentration curve and index

We created a concentration curve to illustrate inequality in ANC service utilization by wealth index and population subgroupings. A concentration curve that falls below the line of equality indicates that wealthy people use the ANC service more. To calibrate the degree and statistical significance of inequality, we used a concentration index, which measures differences in healthcare utilization between the two groups regardless of wealth status [23]. The concentration index ranges from 1 to +1; a value of zero indicates that the health variable is being used equally. When the concentration index is negative, it indicates that health variables are disproportionately concentrated among the poor.

### Decomposition analysis

To analyze the key causes and the amount to which each component contributes to the discrimination in ANC service consumption, a nonlinear logit decomposition model was utilized. The analysis decomposed the total gap in ANC service utilization into two components: endowments and coefficients. Endowments (E) represent differences due to characteristics or endowments between the two groups, such as education, wealth, etc. Coefficients (C) represent differences due to differential effects of endowments or discrimination/bias against one group. Moreover, the raw difference (gap) in ANC service consumption was used to indicate the distinct undecomposed service utilization difference between golden hand and non-golden hand community members. All analyses were judged statistically significant with a *p*-value of less than 0.05 and a confidence range of 95% [25].

## Result

### Sociodemographic characteristics

This study surveyed 1,210 eligible participants, 440 of whom were golden hand community members. The majority of research participants, 516 (42.64%), are in the wealth index’s fifth quintile. The average age of the respondents was 26.64 years, with a standard deviation of +4.76. Most of respondents, 856 (70.50%), identified as Protestant Christians. In terms of residence, half (50.25%) of survey participants lived in rural areas. In terms of occupation, the majority (57.60%) were housewives. When we look at the educational status of the respondents, we show that 38.68% had no formal schooling. And 38.94% of the couples had finished college or higher education, whereas the number of respondents (71.61%) belonged to the Kembata ethnic group (Table 1).

**Table 1:** Sociodemographic characteristics of respondents and their spouses according to groups of women in two communities in the Kembata Tembaro zone, Southern Ethiopia, November 2021.


SOCIOECONOMIC VARIABLES	PARTICIPANT’S CATEGORY		TOTAL PARTICIPANTS

	GOLDEN HAND	NON-GOLDEN HAND	

**Wealth index**			

1st quintile	150 (82.4%)	32 (17.6%)	182 (15.04%)

2nd quintile	104 (82.5%)	22 (17.5%)	126 (10.04%)

3rd quintile	94 (55.0%)	77 (45.0%)	171 (14.13%)

4th quintile	63 (29.3%)	152 (70.7%)	215 (17.77%)

5th quintile	29 (5.6%)	487 (94.4%)	516 (42.64%)

**Residence**			

Urban	399 (65.6%)	209 (34.4)	602 (49.75%)

Rural	41 (6.8%)	561 (93.2%)	608 (50.25%)

**Respondent’s occupation**			

Government employed	16 (3.8%)	408 (96.2%)	697 (57.60%)

Housewife	377 (57.1%)	283 (42.9%)	412 (34.05%)

NGO	12 (48.0%)	13 (52.0%)	69 (5.70%)

Merchant/self-employed	21 (30.9%)	47 (69.1%)	20 (1.65%)

Students	13 (40.6%)	20 (60.3%)	33 (2.73%)

**Spouse’s occupation**			

Government employed	3 (0.8%)	389 (99.2%)	392 (32.59%)

Farmer/housewife	140 (51.5%)	132 (48.5%)	272 (22.52%)

NGO employee	9 (13.01%)	60 (86.99%)	69 (5.74%)

Merchant/self-employed	231 (58.6%)	163 (41.4%)	394 (32.61%)

Student	1(25%)	3 (75%)	4 (0.25%)

Others	64 (87.7%)	9 (12.3%)	73 (6.07%)

**Respondent’s education**			

No formal education	332 (72.2%)	128 (27.8%)	468 (38.68%)

Primary education	90 (48.4%)	96 (51.6%)	187 (15.45%)

Secondary education	9 (5.5%)	156 (94.5%)	165 (13.64%)

College and above	9 (2.3%)	390 (97.7%)	390 (32.23%)


### Utilization of antenatal care services

More than half of the research participants (56.61%) used an ANC service during their previous pregnancy. Most of the non-golden hand community members (80.34 %) used ANC services, but just 12.75% of gold hand community members utilized ANC in their most recent pregnancy. This suggests that the non-golden hand community used ANC services 6.31 times more than the golden hand group. Furthermore, 83.92% of ANC users were in the fifth quantile of the wealth index, 83.55% were urban residents, 86.08% were government employees, and 90.3% of ANC users had finished at least secondary education (Table 2).

**Table 2:** Summary statistics of ANC service utilization of the study participants by socioeconomic variables in the Kembata Tembaro zone, Southern Ethiopia, 2022.


SOCIOECONOMIC VARIABLES	TOTAL NUMBER IN EACH CATEGORY	ANC UTILIZATION

**Community subgroups**		

Golden hand subgroups	440	56 (12.73%)

Non-golden hand subgroups	770	629 (81.69%)

**Wealth index**		

1st quintile	182	42 (23.08%)

2nd quintile	126	22 (17.46%)

3rd quintile	171	67 (39.18%)

4th quintile	215	121 (56.28%)

5th quintile	516	433 (83.91%)

**Residence**		

Urban	602	503 (83.55%)

Rural	608	182 (29.93%)

**Respondent’s occupation**		

Housewife	660	247 (37.42%)

Government employed	424	365 (86.08%)

Merchant/self-employed	68	44 (64.71%)

NGO	25	11 (44.00%)

Students	33	18 (54.55%)

**Respondent’s education**		

No formal education	460	121 (26.30%)

Primary education	186	69 (37.10%)

Secondary education	165	149 (90.30%)

College and above	399	346 (86.72%)


### Knowledge and attitude of study participants toward ANC services

According to modified Bloom’s cutoff points, the overall magnitude of good, medium, and bad knowledge among research participants was 79.87% (95% CI = 77.48 %–82.05 %), 17.44% (95% CI = 15.38%–19.71%), and 2.70% (95% CI = 1.90%–3.80%). The classification was made based on modified Bloom’s cutoff points, and the overall magnitude of positive, neutral, and negative attitude toward the ANC service was 53.72% (95% CI = 50.89%–56.52%), 40.91% (95% CI = 38.16%–43.71%) and 5.37% (95% CI = 4.23%–6.80%), respectively.

### Concentration index and concentration curve showing service utilization inequality

The rate-ratio in Table 3 shows the ratio of service utilization between the richest and poorest wealth quintiles. For ANC service utilization, the richest had 3.64 times higher utilization than the poorest. For ANC knowledge, the richest had 4.02 times higher good knowledge than the poorest. The concentration index shows the degree of socioeconomic inequality in each indicator. Positive values indicate higher utilization/knowledge among richer groups.

**Table 3:** Rate-ratio (rich/poor), concentration indices, standard error, and *p*-value for maternal and reproductive health services in Kembata Tembaro, Southern Ethiopia, 2021.


INDICATORS	RATE-RATIO (RICH/POOR)	COMBINED CONCENTRATION INDEX (CI)	STANDARD ERROR	*P*-VALUE	DIFFERENCE IN CI BETWEEN GOLDEN HAND AND OTHERS	*P*-VALUE FOR TEST OF STATISTICALLY SIGNIFICANT DIFFERENCE (HO: — DIFFERENCE = 0 IF *p* > 0.05)

ANC service utilization*	3.64	0.29	0.014	0.001	0.09	0.0001

Good knowledge of ANC service*	4.02	0.15	0.015	0.001	0.15	0.001

Positive attitude toward ANC service	2.11	0.04	0.02	0.030	-0.01	0.884


* Indicates significant at *p* < 0.05.

ANC service utilization and knowledge both had positive concentration indices showing pro-rich inequality. The concentration index for ANC utilization was 0.29, indicating moderately high pro-rich inequality. The index for knowledge was 0.15, indicating moderately low pro-rich inequality. The attitude index was close to zero (0.04), indicating minimal wealth-related inequality in attitudes. The concentration indices for utilization and knowledge were statistically significant, as evidenced by the low *p*-values. But for attitude, the *p*-value was above 0.05, indicating the concentration index was not statistically different from zero. The difference in concentration indices between golden hand and non-golden hand groups was significant for utilization but not for attitude. This suggests wealth-related inequality in utilization was different between the two groups, but attitude inequality was similar.

Therefore, there were pro-rich inequalities in ANC service utilization and knowledge but not in attitudes. The inequalities were more pronounced among the golden hand group for utilization (Table 3).

**Figure 1:**
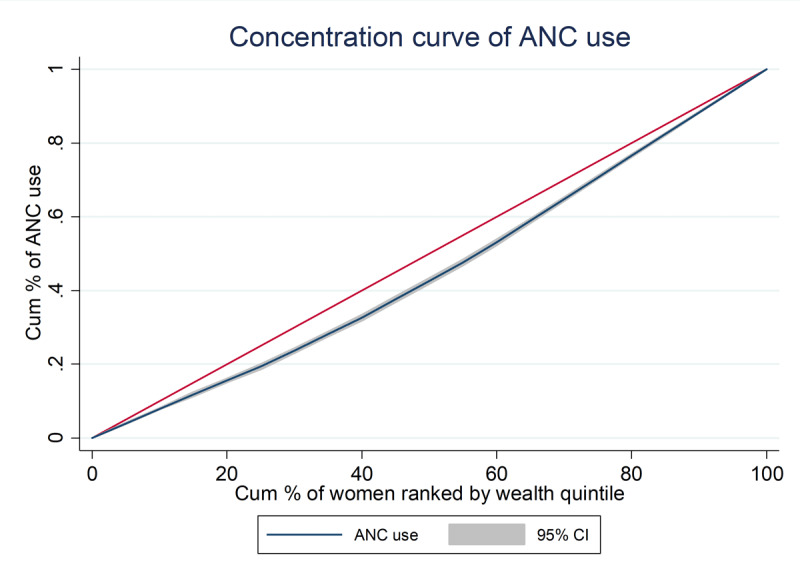
Concentration curve of ANC utilization by wealth quantiles between golden hand and non-golden hand women in the Kembata Tembaro zone, 2021.

**Figure 2:**
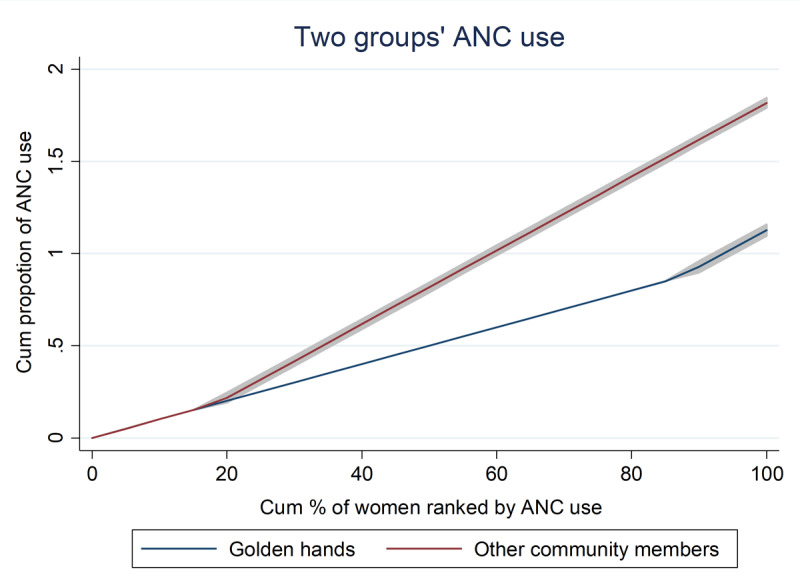
Generalized Lorenz curve showing inequality in ANC service utilization between golden hand and non-golden hand women in the Kembata Tembaro zone, 2021.

The concentration curve graphs the cumulative percentage of the health indicator (y-axis) against the cumulative percentage of the population ranked from poorest to richest (x-axis). The concentration curve for the investigated indicator (ANC) services revealed a pro-rich bias (see Figures 1 and 2 below). The concentration curve of ANC revealed that the services were highly utilized by a wealthy segment of the population. For ANC service utilization, the concentration curve was below the line of equality. This means that as you move from poorest to richest along the x-axis, the cumulative percentage of ANC utilization rises faster than the cumulative share of the population. Therefore, the ANC concentration curve being below the equality line graphically depicts that utilization of ANC services was concentrated or disproportionately higher among the wealthier groups compared to the poorer groups in the study population. This matches the pro-rich inequality found in the quantitative concentration index analysis (Figure 1).

### Generalized Lorenz curve showing inequality in antenatal care service utilization between golden hand and non-golden hand community members

The Lorenz curve for ANC service utilization revealed that the service was lowly utilized by the golden hand community net of characteristics composition. The Lorenz curves depicted below clearly indicate that the service distribution for the golden hand community was more unequal than the service distribution for non-golden hand community members (Figure 2).

### Decomposition analysis for antenatal care service utilization

The total gap in ANC service utilization between the two groups was 0.68, as indicated in Table 4. This means one non-golden hand group had 0.68 higher ANC service utilization than the golden hand group. The raw difference in ANC service consumption in the table below was statistically significant, indicating that there is a distinct undecomposed service utilization difference between golden hand and non-golden hand community members. The gap of 0.689 quantifies how much higher the ANC utilization rate was for one group compared to the other. It provides a numeric measure of the absolute difference in ANC service utilization rates between the two groups. This differential can be presented separately by decomposing it into endowment and discrimination components in order to examine the percentage contribution of each component and forward a clear recommendation on specific components, allowing specific action to be taken on specific components rather than the general recommendation on both components.

**Table 4:** Overall logistic multivariate decomposition analysis of ANC service utilization in the Kembata Tembaro zone, Southern Ethiopia, 2021.


DECOMPOSITION	ANTENATAL CARE (ANC)	

	COEFFICIENT	PCT

Endowments (E)	0.27 (0.17, 0.36)*	39.47

Coefficients (C)	0.41 (0.31, 0.52)**	60.23

Gap (E+C)	0.68 (0.65, 0.72)*	100


*Note*: * *p* < 0.05, ** *p* < 0.01.

The coefficient (discrimination) component is clearly bigger than the endowment component (characteristics), showing that evidence for ANC service use discrimination exists in addition to individual characteristics such as educational achievement, employment, location, and so on. Discrimination explains 60.23% of the lower ANC service consumption by golden hand community members as compared to non-golden community members. Endowment accounts for just 39.47% of the reduced ANC service consumption for the golden hand community subgroups as compared to non-golden hand groupings. The tiny endowment components demonstrate that individual characteristics such as education and other endowment disparities explain a minor portion of the service use differentials among community groupings. However, the discriminating component is substantial, resulting in decreased service consumption by golden hand persons with the same domicile, employment, wealth index, and educational levels as non-golden hand individuals (Table 4).

## Discussion

Inequity between groups in health service utilization are common in developing countries. This study attempted to assess inequities in the utilization of ANC services in the golden hand community in Southern Ethiopia. ANC service is by far utilized more by pregnant women living in urban areas than those living in rural areas. This may be due to increased availability and accessibility of the services in urban areas and to individuals migrating to urban areas from the rural area when they have a strong economic base, which also enhances health-seeking behavior. This finding from the current study is consistent with findings from other inequity studies conducted in Ethiopia in which urban residents were found to utilize the service at a higher rate than rural residents [4], and it is also is in line with findings from a study in a different area [26,27,28,29,30]. Furthermore, ANC service is by far utilized more by educated women than women with no education according to the finding of the current study. This may also be due to the increased intention to utilize ANC service that occurs as a result of advanced educational status. This finding is supported by findings from different studies [28,29,30] and another inequity study in Ethiopia conducted using EDHS data throughout the country [4].

ANC service is utilized more by richer women than poorer women, because as financial capacity increases, health-seeking behavior increases; this is also supported by findings from another study [28]. The rate of ANC service utilization is 4.36 times higher in women in the top wealth quintile than in the bottom quintile. The concentration index was 0.28, which implies that the rich mainly utilize ANC services. This finding is higher than the one reported from Namibia [31], rate-ratio of 1.06 and CI of 0.0130.

Even though they are not considered skilled providers, health extension workers (HEWs) provide preventive and promotive health services. ANC is one of the services provided by HEWs. Less-educated pregnant women, those living in rural areas, and those at the bottom quintile mainly utilize ANC services by HEWs. This could be because HEWs are the first contact with the healthcare system in Ethiopia, especially in rural areas [32,33]. As a result, they can reach the part of the population that is unable to visit health centers or hospitals.

However, the study also found evidence of inequity in the utilization of ANC, and the utilization of at least one antenatal visit was more common among women in the richest quintile [34]. While it is difficult to definitively identify the reasons, the pro-rich inequality in ANC knowledge and utilization may be related to women in the wealthiest quintile having greater access to information about ANC and greater means/opportunity to utilize ANC services. The wealth divide appears to enable richer women to gain more knowledge about ANC and translate that knowledge into higher service utilization compared to the poorest women. Pro-wealth inequality in the services provided in the antenatal program is of great concern, as it implies that the least wealthy pregnant women are not attending the program.

Several studies from varying contexts and populations have reported similar findings of pro-rich inequality and lower utilization of ANC services among disadvantaged minority ethnic groups and those with lower education levels [35,36,37]. For instance, research in Brazil showed improvements in ANC coverage over time but persistent socioeconomic gaps, with women in the lowest wealth quintile having higher inadequate ANC compared to the highest quintile [35]. Several studies from high-income countries like the Unite States have also revealed pronounced racial/ethnic disparities in maternal health services, with Black, Hispanic, and minority women being more likely to face barriers to accessing optimal ANC [37,38]. Furthermore, studies have indicated that there are differences in the utilization of maternal health services between the better-off and poorest populations [24,39,40]. These findings emphasize the need to investigate and assess context-specific causes of varying use of maternal health care if safe motherhood is to become a reality in developing countries [41].

Overall progress toward health-related goals can be reached even if health disparities across socioeconomic groups remain or worsen because certain groups may be left behind or may not benefit to the same extent as the relatively more affluent or privileged [1]. In terms of ANC service knowledge, the rate of having excellent knowledge was greater among economically well-situated women than among the poorest women. This is corroborated by a calculated CI of 0.15, indicating that the wealthier section of the population is more knowledgeable about prenatal care services than the poorest segment. Concerning attitudes about ANC services, the rate of positive attitudes toward ANC services was greater among economically well-situated women than among the poorest women. This is supported by a computed CI of 0.04, revealing that the wealthy segment of the population has a positive attitude toward ANC services compared to the poorest segment.

Women’s understanding of ANC is critical for using ANC services throughout pregnancy. Women’s chances of having a solid understanding of ANC services are therefore a driver for increasing ANC seeking. National-level policies that guarantee targeted ANC services become an inherent component of ANC and other maternity care services and are critical to assisting women in improving their understanding, addressing misconceptions, and removing other healthcare-seeking difficulties while utilizing ANC services [28,42,43]. This suggests that while personal characteristics contribute to low service consumption, discrimination based on living conditions and socioeconomic level accounts for a larger amount of the difference. As a result, the impoverished have higher rates of disease and mortality than the rich, and they typically use healthcare less, even if they have higher needs. These disparities are severe public health issues with social and economic consequences [9,10,11].

## Conclusions

Our ANC service utilization gap decomposition results indicate that a huge service utilization in Ethiopia is out of the ambit of affirmative action policies and here the government should initiate to reserve the right to equal education, employment, earnings, and ANC service utilization opportunity for marginalized sections of the society, like golden hand women and poor, uneducated, and rural people. As a result, policymakers are concerned about huge disparities in prejudice. In terms of endowment differences, improving the economic status and urbanization of marginalized people, job opportunities in government (public and private sectors), and safeguards against discriminatory practices such as norms and many invisible barriers that prevent golden hand women from receiving needed ANC services should be provided to close the huge ANC service utilization gap between golden hand and non-golden hand women.

## Data Accessibility Statement

All data underlying the results are available as part of the article, and no additional source data are required.
